# Bicuspid Aortic Valve Disease: A Comprehensive Review

**DOI:** 10.1155/2012/196037

**Published:** 2012-05-28

**Authors:** Ify Mordi, Nikolaos Tzemos

**Affiliations:** Institute for Cardiovascular Research, British Heart Foundation Glasgow Cardiovascular Research Centre, University of Glasgow, Glasgow G12 8TA, UK

## Abstract

Bicuspid aortic valve is the commonest congenital cardiac abnormality in the general population. This paper article will discuss our current knowledge of the anatomy, pathophysiology, genetics, and clinical aspects of bicuspid aortic valve disease.

## 1. Introduction

Bicuspid aortic valve (BAV) is the commonest congenital cardiac abnormality with an estimated prevalence of 1-2% [1]. It is almost 3 times more common in males than females [2]. Adverse cardiovascular outcomes in patients with BAV are more common than previously thought [3], therefore given its high prevalence it presents potentially a large burden on cardiovascular care.

This paper will discuss our current knowledge of the anatomy, pathophysiology, genetics, and clinical aspects of BAV disease using echocardiographic literature. Only BAV will be discussed in this paper as well as sequelae directly related to this.

### 1.1. Embryology

The definitive fetal cardiac structure is developed by 8 weeks. The semilunar valves form the division of the truncus arteriosus into two separate channels which form the aortic and pulmonary trunks. The channels are created by the fusion of two truncal ridges across the lumen. Small swellings appear on the inferior margins of each of the truncal ridges forming the basis of the adult valve leaflets. In each channel a third swelling occurs opposite the first two which will form the 3rd leaflet. In the normal aortic valve the left and right leaflets of the adult valve are formed from the respective swellings while the posterior leaflet is formed from a swelling in the aortic trunk [4, 5].

The exact pathogenesis of the formation of bicuspid aortic valves is not yet fully understood. It is thought there is certainly a genetic component, especially given the association of BAV with other congenital abnormalities such as coarctation of the aorta. In summary however, the BAV is formed by fusion of the aortic cusps during valvulogenesis.

The pulmonary valve can also be bicuspid, although this is much rarer and is most commonly associated with congenital heart disease such as Tetralogy of Fallot. There have been less than 10 cases reported in the literature of an isolated bicuspid pulmonary valve [6].

### 1.2. Anatomy

**Figure 1:**
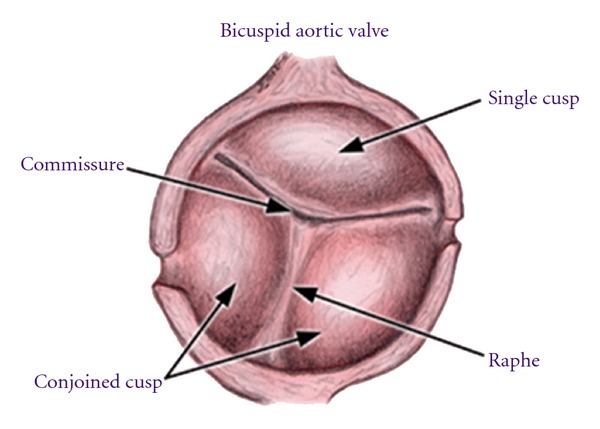
The basic anatomy of the bicuspid aortic valve.

**Figure 2:**
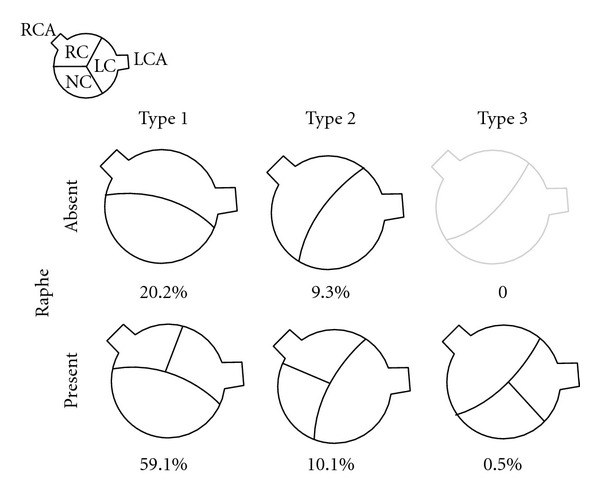
The classification and incidence of bicuspid aortic valves according to site of cusp fusion.

The bicuspid valve is composed of two leaflets, of which one is usually larger [7, 8] (Figure 1). The commonest configuration of the bicuspid valve has the two commissures located in an anteroposterior direction giving left and right cusps while slightly less common is having the commissures located on the right and left sides of the annulus leading to anterior and posterior cusps. The most rare, occurring in less than 1% of patients, is due to fusion of the left and non-coronary cusps. A new classification has identified these as type 1, 2, and 3 bicuspid aortic valves [9] (Figure 2). A raphe is present on the right and anterior cusps respectively, and this can make the valve appear tricuspid on echocardiography. The site of cusp fusion can have effects on the prognosis of BAV [10], with the suggestion that type 1 BAVs are more likely to stenose as adults while type 2 valves will have complications at a younger age. The fused valve leaflet in BAV is actually smaller in area than the total area of two separate leaflets would be if the valve were tricuspid.

As well as valvular lesions there can be several associated nonvalvular lesions. The coronary anatomy can be abnormal. Most patients with BAV disease have a left dominant coronary circulation [8]. This left coronary can arise from the pulmonary artery. The left main can also be up to 50% shorter than in normal in up to 90% of cases [11]. This is an important consideration for any aortic valve surgery.

The commonest abnormality associated with BAV is dilatation of the thoracic aorta, also known as aortopathy. This is thought not only to be due to the altered flow in the aorta, but also due to cellular structural abnormalities including decreased fibrillin, causing smooth muscle cell detachment, and cell death [12].

The other major abnormality found in conjunction with BAV disease is coarctation of the aorta. This occurs in at least 20% of cases and perhaps up to 85% [13, 14]. The presence of coarctation and a poor result from repair can lead to more rapid failure of the valve or aortic dissection. 

### 1.3. Genetics

It is now generally accepted that there is a heritable component to BAV disease. Reports have estimated that there is around a 10% chance of a first degree relative having a bicuspid aortic valve in patients with the disease [15, 16]. A further study indicated a prevalence of almost a quarter in families with more than one member with BAV [17].

The connection of BAV disease and other cardiac abnormalities again suggests that there may be a developmental link. BAV has been found in just over a quarter of patients in a case series of 52 patients with interrupted aortic arch [18].

Mutations in a gene called *NOTCH1*, a transmembrane receptor that has a role in determining cell outcome in organogenesis, were noted in two families with BAV [19]. This seems to be the strongest genetic link discovered yet with further discoveries of missense *NOTCH1* mutations causing impaired Notch signalling [20, 21]. Several other genetic loci have been postulated including chromosomes 18q, 5q, and 13q, though no specific genes have been found.

Recent guidance from the American College of Cardiology/American Heart Association takes into account the genetic component and recommends that all patients with a 1st degree relative with BAV should be evaluated for BAV and aortopathy [22]. No studies have been done as yet however to prove an economic benefit to screening; however recent work has been done to suggest that there is a sufficient pick-up rate of disease if first degree relatives are screened [23]. 

## 2. Diagnosis

Clinical findings are usually limited to auscultation with most patients having an ejection systolic murmur heard loudest at the apex [24]. There may also be signs of aortic stenosis and coarctation of the aorta if associated. The electrocardiogram is usually normal; however there may be signs of left ventricular hypertrophy.

**Figure 3:**
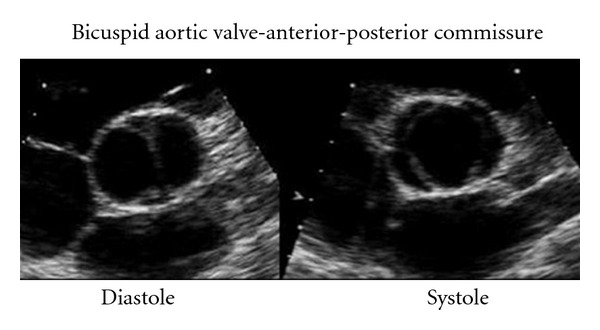
The bicuspid valve in the parasternal short axis view.

The mainstay of diagnosis is echocardiography (transthoracic or transoesophageal) which can provide a definitive diagnosis in the majority of patients (Figure 3). Figures of 92% sensitivity and 96% specificity have been reported when images are adequate [25, 26]. Due to the natural history of BAV to lead to heavily calcified stenotic valves, the utility of echocardiography can be limited [27].

The parasternal short axis view allows for direct visualization of the valve cusps. In this view the normal triangular opening shape is lost, becoming more “fish mouth-”like in appearance, more akin to the mitral valve. This is especially pronounced in systole, as in diastole the raphe can appear similar to a commissure of the third cusp.

A further useful aspect of echocardiography is its ability to identify other cardiac abnormalities including vegetations, systolic dysfunction, and visualisation of part of the aortic root (generally the first 3-4 cm). It is not able however to fully quantify the extent of any aortopathy (whether proximal or distal).

Because of these 2 main limitations, cardiac MRI and CT have been used to augment the diagnostic process. MRI especially will enable views of the valve to be obtained without interference from calcification. It also allows for excellent assessment of the aorta. A recent study of 123 patients with confirmed BAV found that 10% of the patients were misidentified as having a tricuspid valve using transthoracic echo and 28% had a nondiagnostic study, in comparison to 4% being misidentified as having a tricuspid valve by magnetic resonance imaging and 2% having a non-diagnostic study [28]. There is certainly a role for cardiovascular MRI in the assessment of BAV. Additionally, both imaging modalities could be employed to assess the presence and extent of aortopathy making them as excellent surveillance tools.

## 3. Clinical Progression

The natural history of BAV has been evaluated several cohort studies. It is known to be variable and of course somewhat dependent on associated abnormalities. It can range from severe aortic stenosis in childhood to asymptomatic disease until old age. There have indeed been incidental findings of a minimally calcified BAV in patients in their 70s [29]. More commonly however (in around 75% of patients) there is progressive fibrocalcific stenosis of the valve eventually requiring surgery. This usually leads to presentation in middle age—only around 2% of children have clinically significant BAV disease [30].

There have been a couple of studies looking at long-term followup of patients with unoperated BAV. A cohort of 212 asymptomatic patients [31] with BAV (age 32 ± 20 years) were found to have the same 20-year survival rate as the normal population (around 90%) but an increased frequency of cardiac events including aortic valve surgery, ascending aorta surgery and any other cardiovascular surgery. Predictive factors for cardiovascular events were found to be age ≥50 years and valve degeneration at diagnosis while baseline ascending aorta ≥40 mm independently predicted surgery for aorta dilatation.

**Figure 4:**
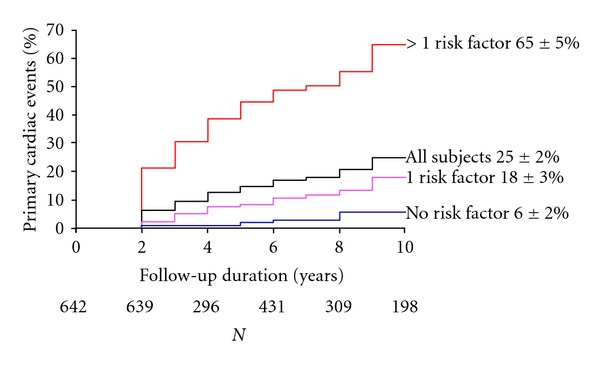
Outcomes in BAV patients (from Tzemos et al. [3]).

Another cohort study [3] looked at outcomes in patients with symptomatic and asymptomatic bicuspid valve disease (mean age 35 year, median 31, range 16–78). 642 patients were followed up for a mean of 9 years, again with a 10-year survival rate similar to the normal population (96%). One or more primary cardiac events occurred in 25% including cardiac death in 3, intervention on aortic valve or ascending aorta in 22%, aortic dissection or aneurysm in 2%, and congestive heart failure requiring hospital admission in 2%. Independent predictors of primary cardiac events were age older than 30 years, moderate or severe aortic stenosis, and moderate or severe aortic regurgitation (Figure 4).

A more recent study has looked at the incidence of aortic complications in 416 BAV patients (mean and median age 35 years, range <1–89) [32]. Incidence of aortic dissection was found to be 1.5% in all patients regardless of the progression of BAV; however this increased markedly in patients aged 50 or older at baseline to 17.4% and even more in those found to have aneurysm formation at baseline to (44.9%). 25-year rate for aortic surgery was 25% and there was a significant burden of progression of disease to cause aortic dissection with 49 of the 384 patients without baseline aneurysms developing them during followup, giving an age-adjusted relative risk of 86.2 and an incidence of 84.9 cases per 10000 patient-years.

The main complications identified in these cohort studies in patients with BAV are aortic stenosis, aortic incompetence, aortopathy/dissection, endocarditis, and sudden death.

### 3.1. Aortic Stenosis

The symptoms of the BAV tend to worsen with increasing stenosis severity, and measurements of the valve orifice. The main symptoms are (exertional) dyspnea, syncope, and chest pain. These patients should be evaluated and managed similarly to patients with tricuspid aortic valve stenosis, but of course the patients will generally present much earlier as described previously.

The foetus can generally survive with severe aortic stenosis due to blood flow through the right side of the heart; however in infancy there is usually a sudden decline in cardiovascular status. One study indicated that children with a valve gradient greater or equal to 50 mmHg had a risk of adverse cardiovascular events of 1.2% per year [33].

In adults with BAV, stenosis occurs by similar methods to the process in patients with tricuspid aortic valves. It is felt to be due to leaflet calcification. It is however more likely to be present in patients by 40 years old. There has been a suggestion that leaflet orientation may be a predictive factor in the rate of valve stenosis [34, 35]; however this was not replicated in the larger studies mentioned earlier [2, 31].

### 3.2. Aortic Incompetence

This is relatively common in BAV and is often independent of aortic stenosis [36, 37]. One cohort of 118 BAV patients found that of 70 patients without aortic stenosis, 28 (40%) had moderate to severe aortic regurgitation. The mechanisms of aortic incompetence in children are usually due to prolapsing cusps, postvalve surgery or endocarditis, while as the patients age dilatation of the ascending aorta can lead to a functionally regurgitant valve. Tzemos et al. [3] however suggested that rates of intervention in BAV patients with solitary aortic incompetence tended to be low. Another important cause of aortic incompetence is myxoid degeneration of the valve. This is where the connective tissue of the valve is replaced by acid mucopolysaccharides disrupting the structural integrity of the valve. One case series included 27 patients with BAV who had pure aortic incompetence—16 of these had severe myxoid degeneration and required earlier intervention than the other 11 (average 40 years versus 52) [38].

### 3.3. Aortopathy/Aortic Dissection

BAV is often associated with dilatation of the aortic root and the ascending aorta [39]. This is otherwise known as aortopathy. This can lead to aneurysm and dissection. The dilatation has been reported during childhood, and it has also been suggested that increased aortic size at baseline is predictive for earlier dilatation and worse outcomes [40, 41]. Aortic size is larger generally in patients with BAV compared to those with normal valves [42]. The most likely risk factor for progression is felt to be age. Aortic root size itself is related to valve morphology and the presence of significant disease [43, 44]; however, a recent study did suggest that while most patients with BAV and ascending aortic aneurysm had severe valve dysfunction, there was a small proportion of patients (5%) who did have aneurysm formation without any aortic valve dysfunction [45].

Many theories have been postulated for the mechanism of BAV aortopathy. For a long time there has been felt to be a genetic component; however there is increasing evidence for a haemodynamic mechanism. It is felt that it is due to defects in the aortic media, such as elastin fragmentation, loss of smooth muscle cells, and an increase in collagen [46–49]. Systemic features have also been noted in BAV patients which may predispose to aneurysm formation including systemic endothelial dysfunction and higher plasma levels of matrix metalloproteinases [50]. Also noted has been an increased amount of wall stress in the ascending aorta [51].

Aortic dissection is a devastating concern in these patients; however the incidence of this has been variable in the studies, from no events [31] and 0.1% [3] in the larger studies, up to 4% in pooled earlier studies [52]. Risk stratification for bicuspid aortic valve and development of aortopathy still has a long way to go as there has so far appeared to be little correlation between echocardiographic and histologic findings and development of aortic disease [53, 54]. Recent advances in echocardiography may help to identify at-risk patients in future [55].

There is still a lot of evidence pointing towards a genetic origin. 4 “important lines of evidence” have been identified for the genetic theory [56]: (1) greater aortic size in patients with BAVs and aortic stenosis compared with those with tricuspid valves and aortic stenosis who are matched for hemodynamic severity [57]; (2) enlarged aortas are found in patients (including children) with BAVs but without any aortic stenosis or aortic regurgitation, compared with age-matched normal controls [58, 59]; (3) studies have demonstrated progressive enlargement of the aorta after aortic valve replacement (AVR) in patients with BAVs [60, 61], (4) studies have demonstrated degeneration of the extracellular matrix of the aorta in patients with BAVs, including elastic fiber fragmentation, increased metalloproteinase expression, decreased expression of tissue inhibitors of metalloproteinases, and smooth muscle cell apoptosis as mentioned previously [50].

### 3.4. Endocarditis

Endocarditis is more common in BAV. The estimated incidence is 0.16% per year in unoperated children and adolescents [62]. In adults the two large case series by Tzemos and Michelena give an incidence of 0.3% and 2% per year, respectively.

Outcomes in BAV patients with infective endocarditis tend to be worse than in those with normal valves. A recent observational study [63] of 310 patients with infective endocarditis found that the 50 patients with BAV were younger at presentation and had a higher incidence of aortic perivalvular abscess. Early surgery was also performed in most of the BAV patients (72%) with similar perioperative mortality to those with tricuspid aortic valves. In-hospital mortality and 5-year survival were also comparable to patients with normal valves.

## 4. Management

The only treatments to offer any sort of curative option are surgical. Medical therapies are to try and alleviate symptoms and slow progression.

### 4.1. Medical

It is generally felt that blood pressure should be aggressively controlled to try and slow the progression of aortopathy. The joint ACC/AHA guidelines suggested use of beta-blockers as first-line therapy in these patients [63]. Extrapolating from patients with aortopathy in Marfan syndrome there is also a suggestion that ACE inhibitors may have a role to play; however the evidence in BAV is still lacking [64].

Of course, concomitant conditions and risk factors should be treated as in the normal population.

### 4.2. Surgical

Indications for valve surgery in patients with BAV are similar to those with tricuspid aortic valves. In children it is usually not practical to do aortic valve replacement as they outgrow the prosthetic valve. Due to the lack of valve calcification in children balloon valvuloplasty is possible and is the management strategy of choice [30]. Studies have shown good followup in both the immediate and mediumterms, with 50% of patients in one series (the majority of whom had BAV) requiring no intervention at 38 months [65].

The 2006 AHA/ACC guidelines also suggest concomitant replacement of the ascending aorta if it is greater than 45 mm in diameter. This has been supported by evidence looking at outcomes in over 200 patients with varying aortic diameters [66]. Estimated 15-year freedom from complications was 86% in patients with an aortic diameter less than 40 mm, dropping down to 81% in those with diameter 40–44 and 43% in patients with a diameter 45 mm or greater.

New techniques of repair such as transcatheter aortic valve implantation have also been reported in BAV [67].

## 5. Conclusion

Bicuspid aortic valve disease is the commonest congenital cardiac abnormality, and because of this it presents a significant burden on cardiac services. Recent cohort studies have given us knowledge of the outcomes of the disease and when to operate; however there is still a need for further evidence for screening and for medical therapies to be evaluated. Also, the role of cardiovascular magnetic resonance as primary imaging tool will continue to enlarge. As our understanding of the pathogenesis of valve degeneration and aortopathy improves this will allow us to identify new targets for treatment.
